# Supplementary material for: DSIF factor Spt5 coordinates transcription, maturation and exoribonucleolysis of RNA polymerase II transcripts
Source: Nat Commun. 2025 Jan 2;16:10. doi: 10.1038/s41467-024-55063-7 (PMC11695829; doi:10.1038/s41467-024-55063-7)
Supplement: Supplementary file 1 — Supplementary Information [file 41467_2024_55063_MOESM1_ESM.pdf]

Supplementary Figure 1

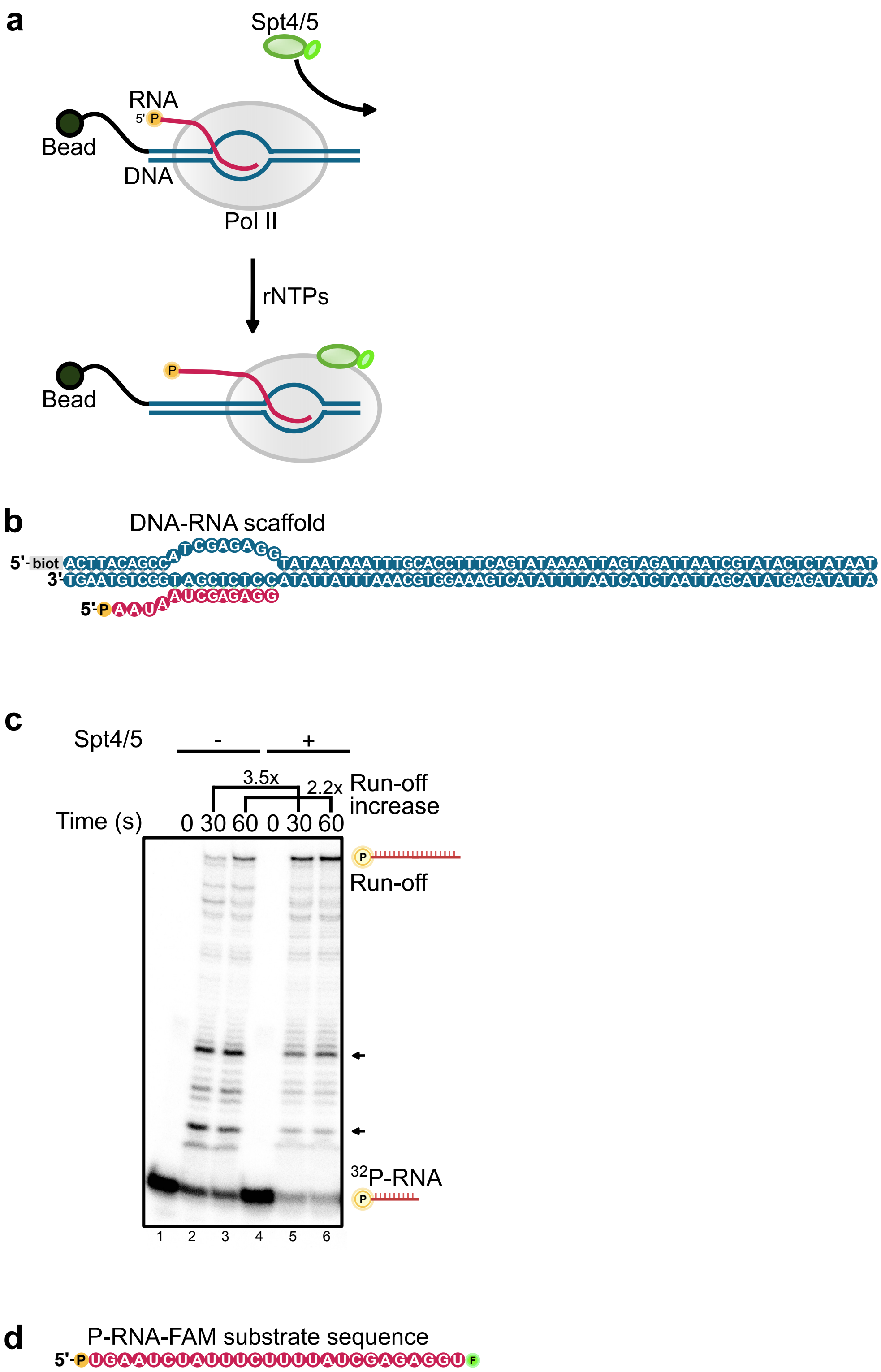

Supplementary Figure 1. The Pol II complex is transcriptionally competent. a The schematic of in vitro transcription assay. Pol II is assembled with RNA-DNA hybrid and immobilised on beads via biotin on non-template DNA strand (scaffold 2, Supplementary Data 2). **b** DNA-RNA scaffold used for in vitro transcription. **c** Spt5 has a stimulatory effect on transcription in vitro. Complexes with or without Spt4/5 were prepared with RNA 5'-labelled with radioactive ^{32}P and transcription was started with ribonucleotides (rNTPs). Samples were collected at the indicated time points and resolved on UREA-PAGE gel. Arrows highlight reduced pausing in the presence of Spt4/5. Relative change of the run-off signal (fully extended RNA) is presented above the gel for the indicated time points. **d** Schematics of 5'-monophosphate-RNA with 3'-FAM fluorescent label (P-RNA-FAM) used for degradation assays.

Supplementary Figure 2

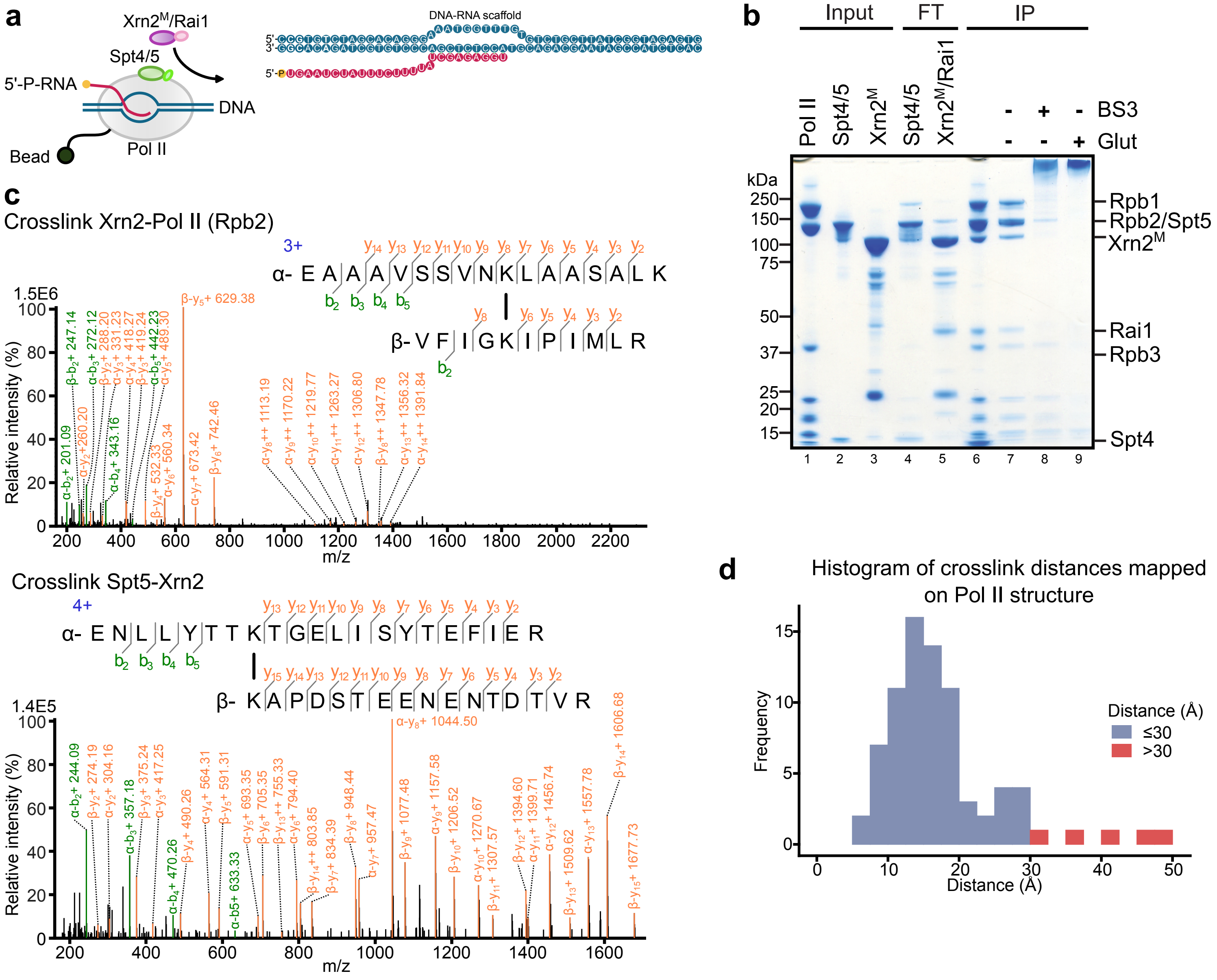

Supplementary Figure 2. Probing Xrn2 interactions with Pol II. a The schematic of in vitro Pol II complex reconstitutions. Pol II was immobilised on α-Flag beads (via Rpb9) and proteins were added in a stepwise manner. Protein excess was washed away, and complexes were eluted with Flag peptide. The nucleic acids scaffold used is shown. **b** Xrn2-Rai1-Spt4/5-Pol II-DNA-RNA complex crosslinked with BS3, or glutaraldehyde (Glut) was analysed by SDS-PAGE gel. FT - refers to the flow-through, IP - immunoprecipitation. **c** Representative spectra for crosslinked peptides between Xrn2-Pol II or Spt5-Xrn2. **d** The distribution of distances between crosslinked peptides mapped on Pol II structure.

Supplementary Figure 3

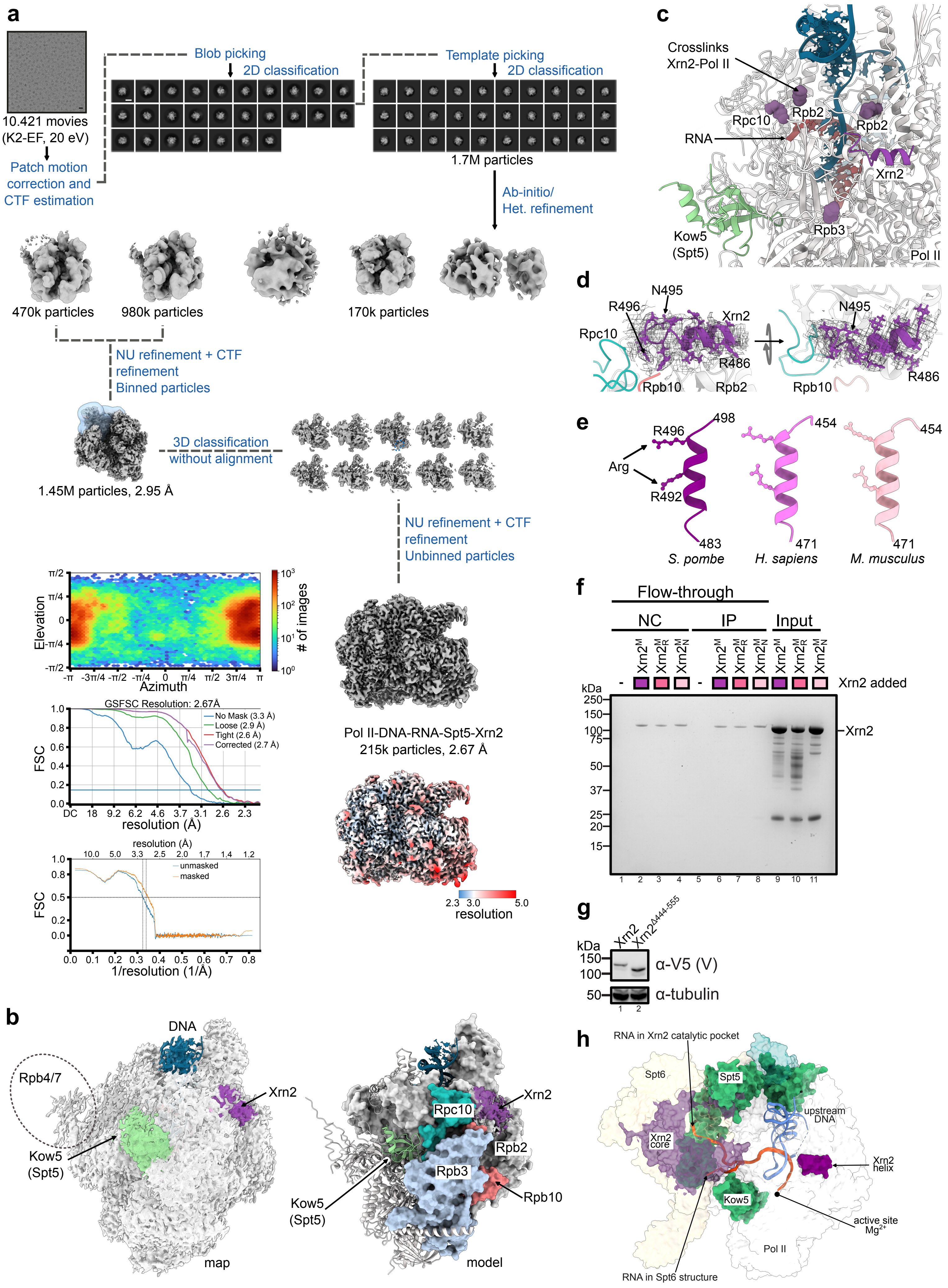

Supplementary Figure 3. Structural analysis of Pol II-Xrn2-Spt5 complex. a Processing of Pol II-Xrn2-Spt5 cryo-EM data. A representative micrograph and 2D classes with scale bars (200 and 100 Å, respectively) are presented together with flowchart and validations. **b** Depiction of the cryo-EM density for the glutaraldehyde crosslinked complex. Areas of Spt5 (Kow5) and Xrn2 peptide binding are highlighted in green and violet, respectively. In the reconstitutions, Rpb4 and Rpb7 were not visible, and their predicted location is marked as an oval. The right panel depicts the model with Rpb2/Rpb3/Rpc10 shown as surfaces. **c** Mapping of the crosslinks between Xrn2 and Pol II on the Pol II-Xrn2-Spt5 structure (compare to Fig. 2c). **d** The electron density map for α-helical loop of Xrn2 is presented as a mesh-like wireframe. **e** AlphaFold prediction of the potential helices that might anchor Xrn2 to Pol II in human and mouse. Two characteristic arginines are shown in the ball-stick representation. **f** SDS-PAGE analysis of the flow-through and input of the in vitro experiment testing binding of Xrn2 mutants to immobilised Pol II-DNA-RNA complex or empty beads (IP and negative control - NC) (related to Fig. 2f). **g** Xrn2 protein levels in whole cell extract for Xrn2 WT or mutant (missing region containing α-helical loop) (compare Figs. 2g and h). Tubulin was used as a loading control. Source data are provided as a Source Data file. **h** Interactions of Spt6 and the Xrn2 catalytic core with Pol II are mutually exclusive. Superimposition of our model (PDB: 8QSZ) with structure featuring elongation complex containing Spt4/5, Elf1, Spt6, Spn1 and Paf1C (PDB: 7XN7). The position of *S. pombe* Xrn2 (PDB: 3FQD) was inferred based on the *S. cerevisiae* Xrn2-Pol II complex (PDB: 8JCH). Arrows indicate the RNA paths in different models.

Supplementary Figure 4

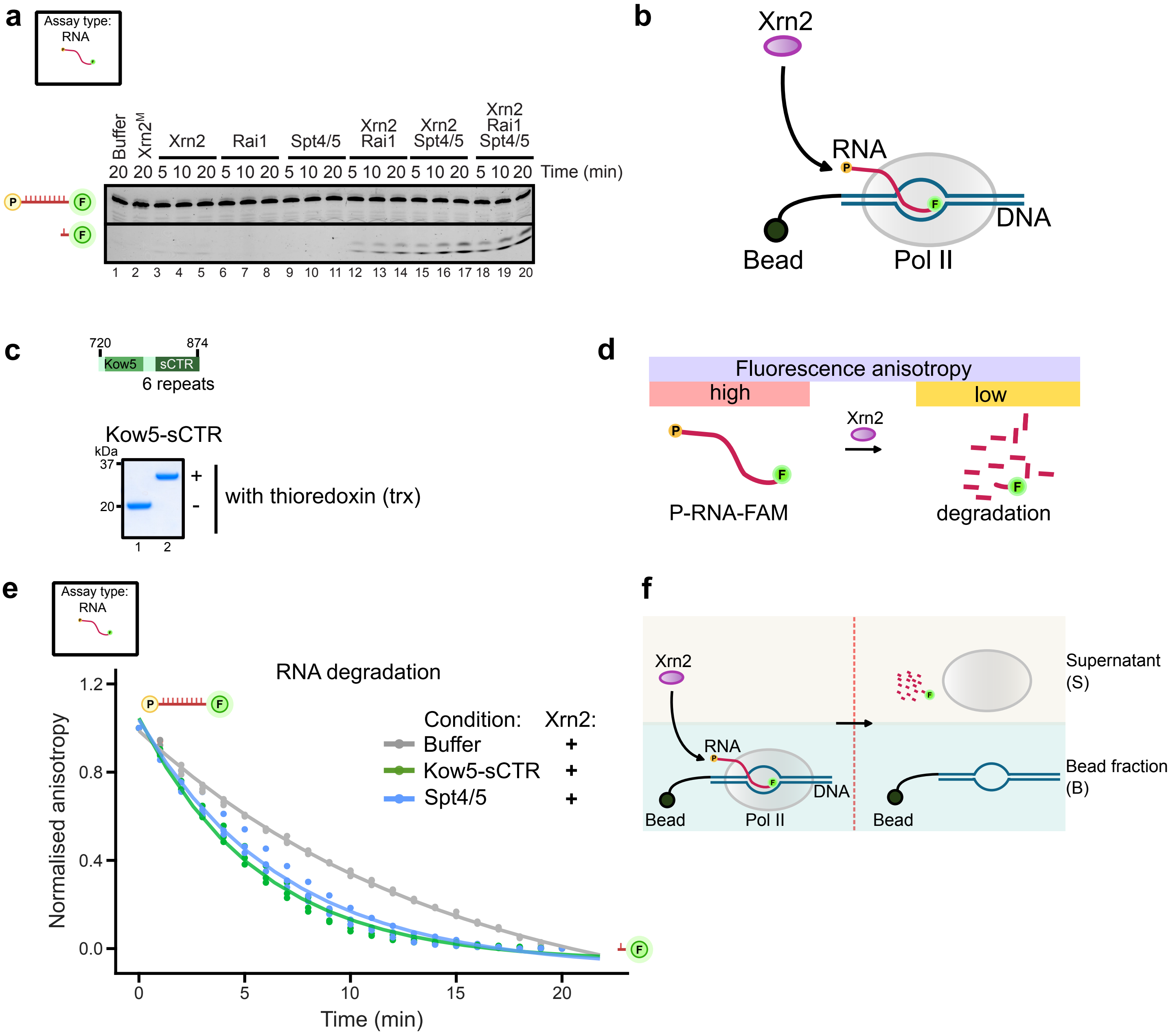

Supplementary Figure 4. Spt5 affects Xrn2 ribonucleolytic activity. a Rai1 and Spt5 can stimulate Xrn2 exoribonucleolytic activity towards 3'-FAM-labelled 5'-monophosphate-RNA substrate (schematic in Supplementary Fig. 1d). Source data are provided as a Source Data file. **b** The schematic diagram for degradation assay with the assembled Pol II complex (scaffold 1a, Supplementary Data 2). When testing the effects of factors (Spt4/5 or Cdk9) on degradation, complexes were washed to remove unbound protein. **c** Purifications of Kow5-sCTR (either as thioredoxin fusions – Supplementary Fig. 4e or His-tag variant protein – Figs. 3b and c). Source data are provided as a Source Data file. **d** The schematic depicting degradation assay based on fluorescence anisotropy. Xrn2 degrades fluorescently labelled RNA, which can be monitored over time. **e** Spt5 stimulates degradation by Xrn2 via fragment containing the Kow5 domain (Kow5-sCTR-Spt5 region 720 to 874 amino acids). Fluorescence polarisation anisotropy assay comparing RNA degradation kinetics of Xrn2 alone, in the presence of Kow5-sCTR or full-length Spt4/5. Constructs used with N-terminal thioredoxin fusion. Source data are provided as a Source Data file. **f** The schematic diagram summarising the in vitro strategy for transcription termination assay. Pol II complex is assembled with nucleic acids and immobilised on streptavidin beads using biotinylated non-template DNA. Xrn2-mediated transcription termination can be evaluated by comparing Pol II amounts in the supernatant or bead (bound fraction).

Supplementary Figure 5

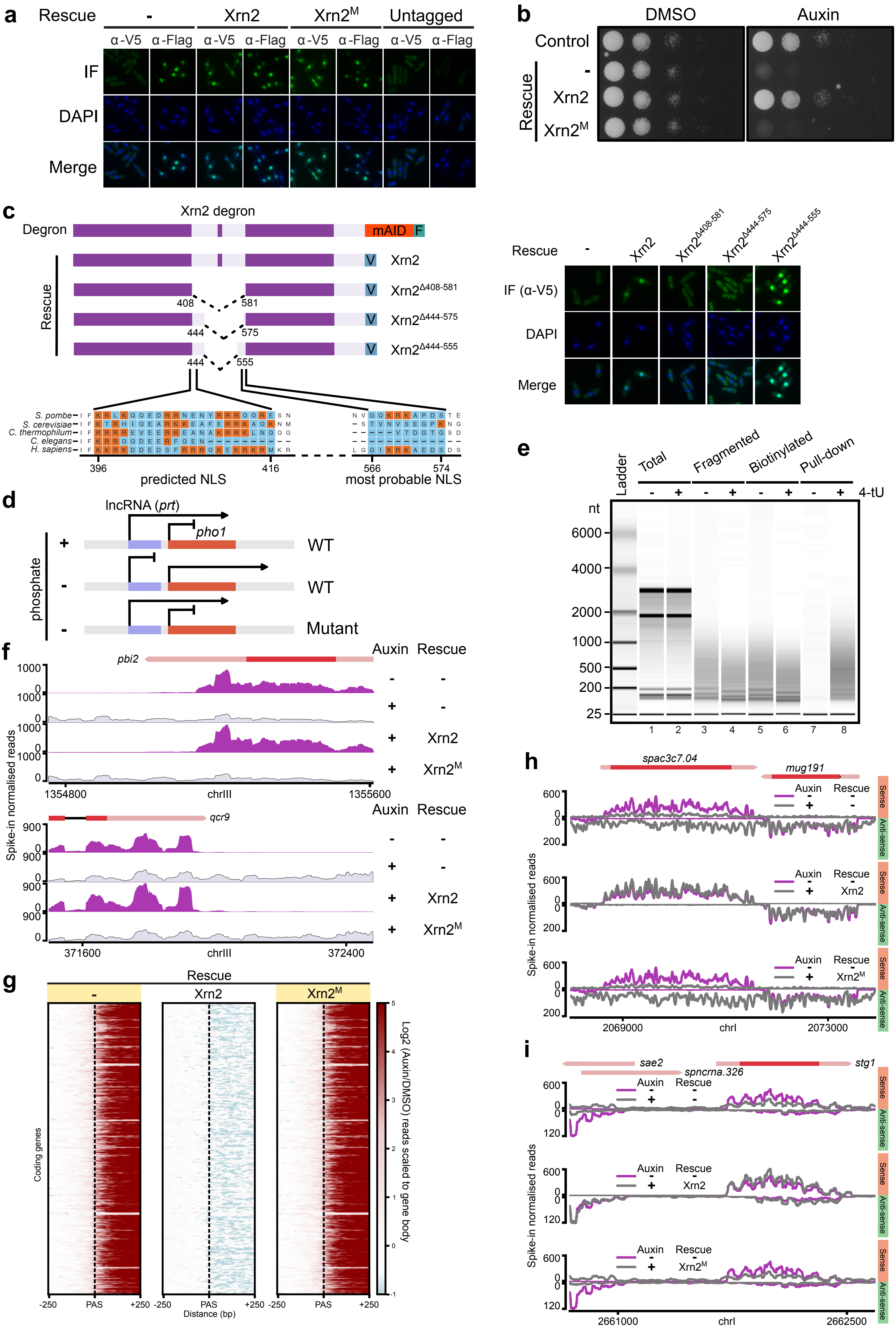

Supplementary Figure 5. Functional analyses of Xrn2 depleted or mutated strains. a Subcellular localisation of Xrn2 mutants (rescue V5-tag and Flag-tag on degron copy). Immunofluorescence (IF), DAPI and merged images are shown. Untagged strain is used to validate the specificity of the antibodies. **b** Growth analysis of strains used in the study grown in the presence or absence of auxin. Expression of two Xrn2 copies does not affect the growth of *S. pombe*. Cells lose their viability after Xrn2 depletion and $Xrn2^{M}$ cannot rescue cell growth defects. **c** Mapping of Xrn2 nuclear localisation signal (NLS). Localisation of different truncation mutants was assessed by immunofluorescence. The predicted NLS is not sufficient to target protein to the nucleus. The most probable NLS is in region 555-575 aa of Xrn2. **d** The schematic diagram explaining phosphatase reporter assay. In the phosphate-rich media, *pho1* gene expression is repressed due to transcription of long non-coding RNA (lncRNA) *prt* that interferes with *pho1* transcription. In the low phosphate conditions, RNA is terminated prematurely to allow expression of the phosphatase (Pho1). If termination is defective (i.e. upon Xrn2 depletion), *prt* transcription interferes with *pho1* expression resulting in low phosphatase activity. **e** Evaluation of TT-seq workflow. RNA is only efficiently pulled down if cells were labelled with 4-thiouracil (4-tU). **f** Representative snapshots demonstrating severe readthrough transcription in the absence of Xrn2 or expression of catalytically inactive Xrn2 mutant (related to Fig. 4d). **g** Genome-wide readthrough affecting coding genes. The region around PAS is presented as a heatmap with a $\log_2$ fold ratio of the indicated treatment to control strain (DMSO treated strain without Xrn2 complementation). Read density is normalised to the gene body (analysis for Spt5 in Fig. 5f). **h** Example genome TT-seq tracks to illustrate gene downregulation by the readthrough from the opposite strand (compare to Fig. 4e, cluster 3 and 1). **i** Locus with genes in tandem orientation showing downregulation of the gene by the upstream readthrough transcription.

Supplementary Figure 6

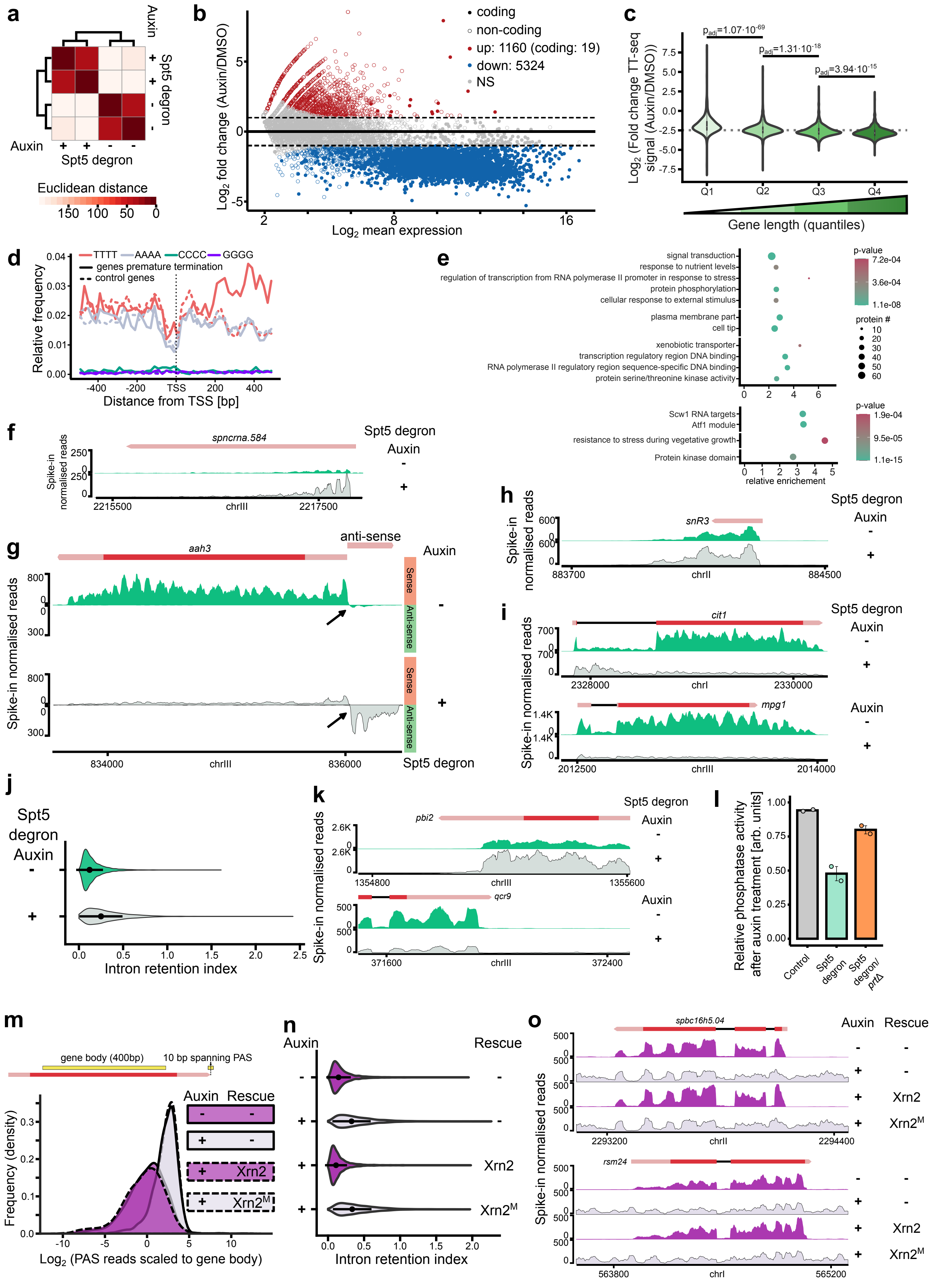

Supplementary Figure 6. Spt5 loss leads to global transcriptional dysregulation. a Heatmap showing Euclidean distances between TT-seq replicates for Spt5 degron (+/- auxin). **b** The MA plot illustrating changes in transcriptome upon loss of Spt5. Spt5 depletion leads to global transcription downregulation. Upregulated genes predominantly belong to the non-coding class. **c** Severity of transcription downregulation correlates with gene length. Statistical analysis was performed using pair-wise Mann-Whitney U test and p-values were adjusted with Benjamini-Hochberg correction. **d** Coding transcription units that show premature termination/attenuation (n=465, compare to Fig. 5d) exhibit higher T content in the TSS region. Runs of nucleotides (at least 4) are shown as a metagene plot. **e** Enrichment gene analysis of coding transcription units that show premature termination/ attenuation (n=465, compare Fig. 5d). A subset of non-redundant categories included, for the full set refer to Supplementary Data 6. **f** Depletion of Spt5 upregulates non-coding RNAs (an example genomic snapshot). **g** Spt5 depletion increases promoter bi-directional anti-sense transcription. **h** Stable non-coding RNAs are affected after Spt5 loss. **i** Spt5 impacts splicing, example genome browser snapshots. **j** Violin plots showing intron retention index before and after Spt5 loss highlighting genome-wide splicing defects. **k** Uncropped genome tracks highlighting transcription readthrough after Spt5 depletion (related to Fig. 5e). **l** Assessment of transcription termination using an endogenous reporter encoding for phosphatase activity (refer to Supplementary Fig. 5d). Phosphatase activity serves as a proxy for termination efficiency. Loss of Spt5 correlates with decreased termination efficiency. Strain, where the long non-coding RNA (*prt* lncRNA) is deleted, serves as a reference for elongation defects after Spt5 loss. Data are presented as mean values +/- SEM (n=2). Source data are provided as a Source Data file. **m** Xrn2 and its catalytic activity are required for functional 3'-end processing (analysis as for Spt5 in Fig. 5h). **n** Violin plots showing intron retention index in Xrn2-degron strains before and after auxin treatment. **o** The readthrough generated after the loss of Xrn2 activity is not properly spliced.
