# Supplementary material for: DSIF factor Spt5 coordinates transcription, maturation and exoribonucleolysis of RNA polymerase II transcripts
Source: Nat Commun. 2025 Jan 2;16:10. doi: 10.1038/s41467-024-55063-7 (PMC11695829; doi:10.1038/s41467-024-55063-7)
Supplement: Supplementary file 2 — Description of Additional Supplementary Files [file 41467_2024_55063_MOESM2_ESM.pdf]

Description of Additional Supplementary Files

File Name: Supplementary Data 1

Description: Proteins found in Spt5-3xFLAG purifications (or mutants). List of proteins captured by Mass-Spectrometry analysis.

File Name: Supplementary Data 2

Description: List of oligonucleotides used for in vitro experiments and RT-PCR.

File Name: Supplementary Data 3

Description: List of BS3 crosslinked peptides within Pol II - Spt5/4 - Xrn2/Rai1 complex.

File Name: Supplementary Data 4

Description: The statistics of cryo-EM structure.

File Name: Supplementary Data 5

Description: The statistical analysis of phosphatase reporter assay activity for Xrn2 depletion.

File Name: Supplementary Data 6

Description: Gene enrichment analysis for transcripts with premature termination.

File Name: Supplementary Data 7

Description: Strains and plasmids used in this study.
