# Supplementary material for: DSIF factor Spt5 coordinates transcription, maturation and exoribonucleolysis of RNA polymerase II transcripts
Source: Nat Commun. 2025 Jan 2;16:10. doi: 10.1038/s41467-024-55063-7 (PMC11695829; doi:10.1038/s41467-024-55063-7)
Supplement: Supplementary file 10 — Reporting Summary [file 41467_2024_55063_MOESM10_ESM.pdf]

nature portfolio

Corresponding author(s): Lidia Vasiljeva, Krzysztof Kus

Last updated by author(s): Oct 2, 2024

Reporting Summary

Nature Portfolio wishes to improve the reproducibility of the work that we publish. This form provides structure for consistency and transparency in reporting. For further information on Nature Portfolio policies, see our Editorial Policies and the Editorial Policy Checklist.

Statistics

For all statistical analyses, confirm that the following items are present in the figure legend, table legend, main text, or Methods section.

n/a	Confirmed	
☐	☒	The exact sample size (*n*) for each experimental group/condition, given as a discrete number and unit of measurement
☐	☒	A statement on whether measurements were taken from distinct samples or whether the same sample was measured repeatedly
☐	☒	The statistical test(s) used AND whether they are one- or two-sided<br>*Only common tests should be described solely by name; describe more complex techniques in the Methods section.*
☒	☐	A description of all covariates tested
☐	☒	A description of any assumptions or corrections, such as tests of normality and adjustment for multiple comparisons
☐	☒	A full description of the statistical parameters including central tendency (e.g. means) or other basic estimates (e.g. regression coefficient) AND variation (e.g. standard deviation) or associated estimates of uncertainty (e.g. confidence intervals)
☐	☒	For null hypothesis testing, the test statistic (e.g. *F*, *t*, *r*) with confidence intervals, effect sizes, degrees of freedom and *P* value noted<br>*Give P values as exact values whenever suitable.*
☒	☐	For Bayesian analysis, information on the choice of priors and Markov chain Monte Carlo settings
☒	☐	For hierarchical and complex designs, identification of the appropriate level for tests and full reporting of outcomes
☒	☐	Estimates of effect sizes (e.g. Cohen's *d*, Pearson's *r*), indicating how they were calculated

Our web collection on statistics for biologists contains articles on many of the points above.

Software and code

Policy information about availability of computer code

Data collection	SerialEM v3.8
Data analysis	fastp 0.20.0<br>STAR 2.7.3<br>deeptools 3.5.x<br>samtools 1.x<br>cryoSPARC V-3.X<br>RELION 3.1<br>python 3.8<br>R studio 4.x<br>DESeq2<br>pLink software version 2.3.9

For manuscripts utilizing custom algorithms or software that are central to the research but not yet described in published literature, software must be made available to editors and reviewers. We strongly encourage code deposition in a community repository (e.g. GitHub). See the Nature Portfolio guidelines for submitting code & software for further information.

Data

Policy information about availability of data

All manuscripts must include a data availability statement. This statement should provide the following information, where applicable:
- Accession codes, unique identifiers, or web links for publicly available datasets
- A description of any restrictions on data availability
- For clinical datasets or third party data, please ensure that the statement adheres to our policy

The model and map for the structure have been deposited in the PDB: 8QSZ and EMDB: EMD-18643. TT-seq and ChIP-seq data have been deposited in Gene Expression Omnibus under GSE244546 and GSE273510, respectively.

Research involving human participants, their data, or biological material

Policy information about studies with human participants or human data. See also policy information about sex, gender (identity/presentation), and sexual orientation and race, ethnicity and racism.

Reporting on sex and gender	N/A
Reporting on race, ethnicity, or other socially relevant groupings	N/A
Population characteristics	N/A
Recruitment	N/A
Ethics oversight	N/A

Note that full information on the approval of the study protocol must also be provided in the manuscript.

Field-specific reporting

Please select the one below that is the best fit for your research. If you are not sure, read the appropriate sections before making your selection.

☒ Life sciences ☐ Behavioural & social sciences ☐ Ecological, evolutionary & environmental sciences

For a reference copy of the document with all sections, see nature.com/documents/nr-reporting-summary-flat.pdf

Life sciences study design

All studies must disclose on these points even when the disclosure is negative.

Sample size	Not relevant to this study
Data exclusions	No data exclusion
Replication	Experiments performed at least twice
Randomization	Not relevant to the study
Blinding	Not relevant to the study

Reporting for specific materials, systems and methods

We require information from authors about some types of materials, experimental systems and methods used in many studies. Here, indicate whether each material, system or method listed is relevant to your study. If you are not sure if a list item applies to your research, read the appropriate section before selecting a response.

April 2023

Materials & experimental systems

n/a	Involved in the study
☐	☒ Antibodies
☒	☐ Eukaryotic cell lines
☒	☐ Palaeontology and archaeology
☒	☐ Animals and other organisms
☒	☐ Clinical data
☒	☐ Dual use research of concern
☒	☐ Plants

Methods

n/a	Involved in the study
☐	☒ ChIP-seq
☒	☐ Flow cytometry
☒	☐ MRI-based neuroimaging

Antibodies

Antibodies used

mouse monoclonal anti-Rpb1 (Pol II) 8WG16 antibody, cat. GTX20817, lot 822400632, 822401948, GeneTex;
mouse monoclonal anti-V5-tag antibody, MCA1360, lot 158243, Bio-Rad;
rat monoclonal anti-alpha-tubulin antibody, ab6160, Abcam;
mouse monoclonal anti-Flag M2-Peroxidase (HRP) antibody, A8592, Sigma-Aldrich;
mouse monoclonal anti-V5-tag antibody,E-AB-20010, Elabscience;
mouse monoclonal anti-V5-Peroxidase antibody, V226, Sigma-Aldrich;
rabbit polyclonal anti-histone H3 Antibody, CT, pan, 07-690, Sigma-Aldrich

Validation

N/A

Plants

Seed stocks

N/A

Novel plant genotypes

N/A

Authentication

N/A

ChIP-seq

Data deposition

☒ Confirm that both raw and final processed data have been deposited in a public database such as GEO.

☐ Confirm that you have deposited or provided access to graph files (e.g. BED files) for the called peaks.

Data access links
May remain private before publication.

https://www.ncbi.nlm.nih.gov/geo/query/acc.cgi?&acc=GSE273510

Files in database submission

GSM8430350 Input, auxin, replicate1
GSM8430351 Input, auxin, replicate2
GSM8430352 Input, DMSO, replicate1
GSM8430353 Input, DMSO, replicate2
GSM8430354 PolII ChIP, auxin, replicate1
GSM8430355 PolII ChIP, auxin, replicate2
GSM8430356 PolII ChIP, DMSO, replicate1
GSM8430357 PolII ChIP, DMSO, replicate2
GSM8430358 PolII ChIP, DMSO, Xrn2-444-555Δ, replicate1
GSM8430359 PolII ChIP, DMSO, Xrn2-444-555Δ, replicate2
GSM8430360 Xrn2 ChIP, auxin, replicate1
GSM8430361 Xrn2 ChIP, auxin, replicate2
GSM8430362 Xrn2 ChIP, DMSO, replicate1
GSM8430363 Xrn2 ChIP, DMSO, replicate2
GSM8430364 Xrn2-444-555Δ ChIP, DMSO, replicate1
GSM8430365 Xrn2-444-555Δ ChIP, DMSO, replicate2

Genome browser session
(e.g. UCSC)

bigwigs are provided in GEO, token: irkrwakqxzmldoj

Methodology

Replicates

2

Sequencing depth

pair-end
2x40
Sample Total reads Unique reads
Input, DMSO, replicate1 53631285 40871658
Input, DMSO, replicate2 57847393 43939320
Input, auxin, replicate1 56241048 41770525
Input, auxin, replicate2 55729091 40803280
PolII ChIP, DMSO, replicate1 67998509 62684830
PolII ChIP, DMSO, replicate2 48991232 45176846
PolII ChIP, DMSO,Xrn2-444-555Δ, replicate1 70555198 64641289
PolII ChIP, DMSO,Xrn2-444-555Δ, replicate2 67264418 61014006
PolII ChIP, auxin, replicate1 73527169 65498347
PolII ChIP, auxin, replicate2 70532284 62897414
Xrn2 ChIP, DMSO, replicate1 69221263 50990234
Xrn2 ChIP, DMSO, replicate2 79026247 58474394
Xrn2 ChIP, auxin, replicate1 81706574 59321427
Xrn2 ChIP, auxin, replicate2 76625863 53960720
Xrn2-444-555Δ ChIP, DMSO, replicate1 66989068 48252714
Xrn2-444-555Δ ChIP,DMSO, replicate2 81493619 57745819

Antibodies

Rpb1 (Pol II) 8WG16, cat. GTX20817, lot 822400632, 822401948, GeneTex
anti-V5, V5-tag, MCA1360, lot 158243, Bio-Rad (used for Xrn2 tagged with V5)

Peak calling parameters

N/A

Data quality

No peak calling used. Input was sequenced and subtracted from ChIP signal. Two replicates were concordant.

Software

fastp 0.20.0
STAR 2.7.3
deeptools 3.5.x
samtools 1.x
